# Transvaginal Ultrasound vs. Magnetic Resonance Imaging (MRI) Value in Endometriosis Diagnosis

**DOI:** 10.3390/diagnostics12071767

**Published:** 2022-07-21

**Authors:** Alexandra Baușic, Ciprian Coroleucă, Cătălin Coroleucă, Diana Comandașu, Roxana Matasariu, Andrei Manu, Francesca Frîncu, Claudia Mehedințu, Elvira Brătilă

**Affiliations:** 1 Department of Obstetrics and Gynecology, “Carol Davila” University of Medicine and Pharmacy, 020021 Bucharest, Romania; alexandrabausic@gmail.com (A.B.); cip_coroleuca@yahoo.com (C.C.); diana.comandasu@yahoo.com (D.C.); francesca.frincu@drd.umfcd.ro (F.F.); claudia.mehedintu@umfcd.ro (C.M.); 2 Department of Obstetrics and Gynecology, “Prof. Dr. Panait Sîrbu” Obstetrics and Gynecology Hospital, 060251 Bucharest, Romania; ccoroleuca@yahoo.com (C.C.); andrei.manu16@yahoo.co.uk (A.M.); 3 Department of Mother and Child “Grigore T. Popa”, University of Medicine and Pharmacy, 700115 Iași, Romania; roxanamatasariu@yahoo.com

**Keywords:** endometriosis, transvaginal ultrasound, magnetic resonance imaging, diagnosis

## Abstract

(1) Background: Endometriosis is a widespread gynecological condition that causes chronic pelvic discomfort, dysmenorrhea, infertility, and impaired quality of life in women of reproductive age. Clinical examination, transvaginal ultrasonography (TVS), and magnetic resonance imaging (MRI) are significant preoperative non-invasive diagnosis procedures for the accurate assessment of endometriosis. Although TVS is used as the primary line for diagnosis, MRI is commonly utilized to achieve a better anatomical overview of the entire pelvic organs. The aim of this systematic review article is to thoroughly summarize the research on various endometriosis diagnosis methods that are less invasive. (2) Methods: To find relevant studies, we examined electronic databases, such as MEDLINE/PubMed, Cochrane, and Google Scholar, choosing 70 papers as references. (3) Results: The findings indicate that various approaches can contribute to diagnosis in different ways, depending on the type of endometriosis. For patients suspected of having deep pelvic endometriosis, transvaginal sonography should be the first line of diagnosis. Endometriosis cysts are better diagnosed with TVS, whereas torus, uterosacral ligaments, intestine, and bladder endometriosis lesions are best diagnosed using MRI. When it comes to detecting intestine or rectal nodules, as well as rectovaginal septum nodules, MRI should be the imaging tool of choice. (4) Conclusions: When diagnosing DE (deep infiltrative endometriosis), the examiner’s experience is the most important criterion to consider. In the diagnosis of endometriosis, expert-guided TVS is more accurate than routine pelvic ultrasound, especially in the deep infiltrative form. For optimal treatment and surgical planning, accurate preoperative deep infiltrative endometriosis diagnosis is essential, especially because it requires a multidisciplinary approach.

## 1. Introduction

Endometriosis is a painful chronic gynecological condition that causes infertility and pelvic pain [1]. Although it is a benign condition, it is also a significant medical, social, and economic ongoing issue to the accompanying symptoms and chronic character.

Endometriosis has also been linked to more severe pregnancy outcomes (preeclampsia, preterm delivery, placental conditions) [2]. Endometriosis is an estrogen-dependent disorder characterized by the presence of ectopic tissue (endometrial glandular cells and stroma) outside the uterus [1]. The ectopic endometrium is functionally similar to the eutopic endometrium [1,2,3]. About ten percent of women of childbearing age are negatively affected by this condition, which results in infertility in 30 to 50 percent of situations [3].

Dysmenorrhea (painful periods), dyspareunia (painful intercourse), persistent pelvic pain, and infertility are all symptoms of endometriosis [3]. The exact pathological mechanisms of endometriosis are unclear; however, this condition may appear as a result of the dissemination of the eutopic endometrial tissue to ectopic sites [4].

Pelvic endometriosis is characterized by endometrial cells localized in the pelvic cavity, including the peritoneum, the pelvic organs, and inside the pouch of Douglas (POD) [3]. Endometriomas, commonly known as ovarian endometriotic cysts, are ovarian lesions that appear as masses of various diameters and are encircled by endometrial tissue [5].

The small superficial endometriosis lesions infiltrate the pelvic organs at a depth of less than 5 mm beneath the peritoneum’s surface, and macroscopically differ depending on their activity, ranging from blue-black “powder burns” to red or white flat areas [6]. These lesions are best observed laparoscopically along the peritoneal lining or the surface of the ovary [6].

Deep infiltrating endometriosis (DE) is described as endometriotic lesions that infiltrate the pelvic organs at a depth of 5 mm or more beneath the peritoneum’s surface. Endometriotic nodules can be detected in multiple locations, including the pelvic peritoneum, the anterior and posterior pelvic compartments, or both [6,7]. The uterosacral ligaments (USL), POD, and bowel are the most common sites of DE, which primarily occur below the rectosigmoid junction [3].

Extrapelvic endometriosis may appear in lung, liver, pancreas, and operative scars, accompanied by specific symptom phenotypes [7]. Although each form of endometriosis represents a distinct clinical entity, different types of endometriosis can coexist in the same patient [4,7].

Despite considerable research regarding endometriosis diagnosis, there is still doubt as to whether an MRI is redundant and whether transvaginal ultrasound examination (TVS) should be the only diagnostic method.

This review analyzes the feasibility of accurately diagnosing DE using imaging methods before the surgical treatment, insisting on the TVS and magnetic resonance imaging (MRI) comparison. We aimed to answer the concerns about whether clinical examination and transvaginal ultrasound are sufficient, and when the MRI examination is required. The objective of this review is to compile existing knowledge regarding the reliability of endometriosis diagnostic procedures, and to assess the research’s advantages and disadvantages.

## 2. Materials and Methods

We checked for relevant articles in The Cochrane Library, The Wiley Online Library, and PUBMED. The terms “deep infiltrating endometriosis”, “clinical signs”, “physical examination”, “transvaginal ultrasound”, and “magnetic resonance imaging” were used in combination. In addition, review articles and guidelines from the Royal College of Obstetricians and Gynecologists in the United Kingdom and the American Society for Reproductive Medicine (ASRM) were consulted.

**Figure 1:**
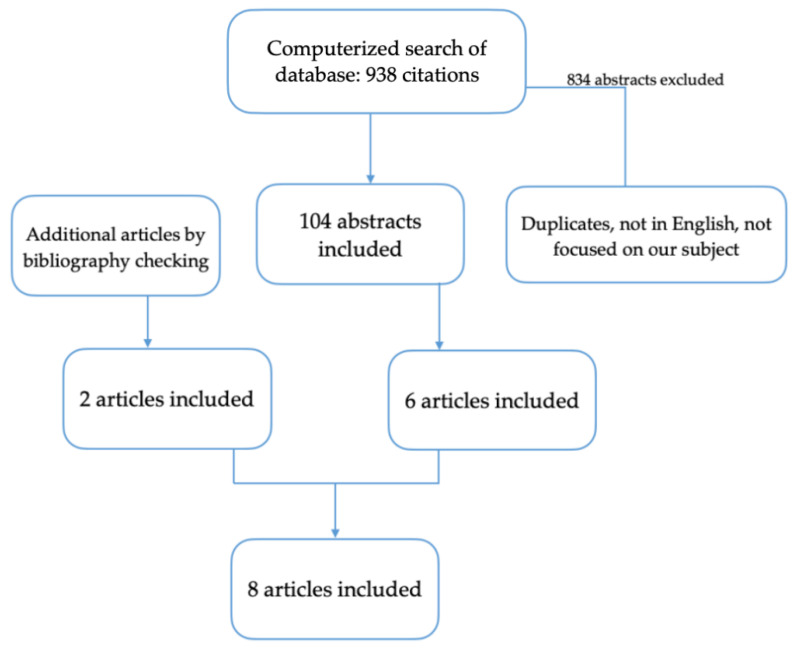
Flow diagram of the search process.

We have chosen to use the results from eight studies to compare the accuracy of the two imagistic methods in diagnosing endometriosis. The eight included studies [8,9,10,11,12,13,14,15] have been published from 2009 to 2019 and reported on 893 patients (Figure 1). The homogeneous methodology and techniques used in the chosen studies, as opposed to earlier research, are what determine our study’s significance.

## 3. Results

### 3.1. Diagnosis Methods

Until recently, laparoscopic exploration with biopsy of intraperitoneal cavity lesions has been considered the gold standard diagnostic method due to direct insight into the endometriosis lesions [16]. However, it is not an unhazardous procedure, and may fail to recognize retroperitoneal elements such as nerve fibers and ureters, or visualize endometriotic lesions [16,17]. A definitive diagnostic can only be established by the histological interpretation of lesions removed during surgery.

Diagnostic laparoscopy is expensive and implies surgical risks; therefore, diagnostic imaging methods have been discussed to see if they are reliable in detecting endometriosis without the need for an intervention [18]. A reliable imaging method can contribute to an accurate assessment of the disease’s degree, reducing the number of procedures, or limiting the number of patients who need surgery to those who are most likely to have DE [18].

Furthermore, if imaging tests can accurately identify the location of DE lesions, surgeons might have the evidence they need to prepare and enhance their surgical strategy.

Since there is a limited association between symptoms and lesion severity, some authors recommend a fundamental change to a more specific clinical strategy, integrating the symptomatology, imaging data, and symptomatic treatment outcome before surgical confirmation [19].

Patients with dysmenorrhea, non-cyclical pelvic pain, dyspareunia, infertility, dyschezia, dysuria, hematuria, or rectal bleeding should be investigated for an endometriosis diagnosis [20]. All patients with endometriosis suspicion should undergo a clinical examination [20].

Multiple cases with endometriosis may remain completely asymptomatic. Patients at risk should be identified with a detailed clinical history and imaging diagnosis, as a diagnosis based purely on the severity of the symptoms can be misleading [21].

Bimanual vaginal examination is positive and supports the diagnosis when encountering a fixed retroverted uterus, pelvic discomfort, tender and shortened uterosacral ligaments, and adnexal masses (palpable nodule, thickened area, or a palpable cystic expansion) [22]. If deep-infiltrating nodules are palpated on the rectovaginal wall or seen in the posterior vaginal fornix during clinical examination, diagnosis is more straightforward [22,23]. Clinical examinations performed during menstruation are the most reliable for detecting deep-infiltrating nodules; however, patient acceptance may be a concern [23].

As far as we know, scarce research has been focused on the pelvic exam’s capacity to predict endometriosis diagnosis. Despite its low accuracy, a pelvic examination is nevertheless a significant step in the initial diagnosis of DE, providing a better understanding of the disease extent, which is important for planning therapeutical approaches [22].

DE can be diagnosed using a variety of noninvasive imaging techniques, including magnetic resonance imaging (MRI), transvaginal ultrasonography (TVS), transrectal sonography (TRS), and 3D ultrasound [8]. Research has focused on the predictive diagnostic value of these techniques. All these modalities rely on the interpreter’s experience and expertise for their performance and interpretation [17].

TVS is the most accessible technique of diagnosis in endometriosis [17]. TVS is frequently the first diagnostic imaging modality in symptomatic DE patients due to its cost-effectiveness and accessibility [8,24]. It allows to identify the difference between endometriotic implants and ovarian cysts, and to exclude other causes of pelvic pain syndrome [10]. Patients may experience severe discomfort when the transductor is pressed against the endometriotic nodule [24].

MRI is a noninvasive, but costly, method of diagnosing DIE that can scan the entire peritoneal cavity, providing more accurate information on the disease’s extension and localization [8,25]. Timely screening of endometriosis can enable appropriate pharmacologic utilization and surgical interventions to manage symptoms and enhance patients’ long-term outcomes while also lowering expenses [25].

The two imaging techniques are also widely used to diagnose endometrial cancer, as well as other gynecological conditions. Due to its capacity to distinguish it from benign disorders such as leiomyomas or adenomyosis, MRI is recommended as the tool of choice in this setting [26].

#### 3.1.1. Ultrasonography Overview

The accuracy of transvaginal sonography in identifying DE is higher in intestinal and bladder endometriosis, and lower in vaginal, uterosacral, and rectovaginal septum lesions, according to the findings of Bazot M et al. [27]. The sonographic observations contributed to the morphological criteria classification for diagnosing endometriotic lesions [27].

Endometriomas are easier to be detected by pelvic examination or TVS than other types of endometriosis; nevertheless, it might be hard to differentiate between endometriomas and ovarian malignancies [28]. Kupfer et al. defined the appearance of diffuse low-level echoes within cysts as endometriotic involvement of the ovaries [28].

Homogeneous echoes, often known as ground-glass look, are the characteristic ultrasound aspect of these cysts due to hemorrhagic content, and they do not usually show any vascular echo with a Doppler flow scanner [29]. The “kissing ovaries” sign, with both the ovaries adherent to the posterior uterine wall, is generally linked with bilateral ovarian endometriosis cysts, and can indicate the existence of pelvic adhesions and DE [30,31].

The findings of G. Hudelist et al. define the sonographic diagnostic criteria for different types of endometriotic lesions in the pelvic area [9].

The anterior pelvic compartment contains the urinary bladder, the urethra, the vesicouterine pouch, and the round ligaments [20]. If a hypoechogenic nodule with or without cystic characteristics is visible on the posterior wall of the urinary bladder, endometriosis of the bladder is hypothesized [32].

In a paracervical nodule appearance on TVS, ureteral infiltration should be considered. DE can cause ureteral blockage, which can progress to hydronephrosis and gradual kidney failure; therefore, a genitourinary ultrasound exam followed by specialized renal dysfunction examinations may be required [33].

The uterus and the adnexa are located in the center pelvic compartment, whereas the posterior compartment hosts the Douglas pouch, the uterine torus, the rectovaginal septum, the uterosacral ligaments, and the rectosigmoid [33].

Abnormal hypoechogenic linear thickening and/or the hypoechogenic cystic or non-cystic lesion inside the posterior vaginal wall are categorized as vaginal involvement [9]. The rectovaginal space may have endometriosis implants when hypoechogenic nodules or cysts are visualized [9].

When the uterus, ovaries, tubes, and rectosigmoid colon form a common block with the disappearance of the peritoneal structures, and peritoneal borders are only partially recognized, the obliteration of the posterior pelvic pouch is deemed complete [32]. The “kissing ovaries” TVS sign suggests the attachment of the ovaries to the pouch of Douglas through pelvic adhesions [34]. This observation was linked to higher laparoscopic classification severity scores (#Enzian and rASRM) and longer surgical duration because of more widespread disease in the pouch of Douglas and pelvic area [30,34,35,36,37,38].

According to Bazot’s criteria, the uterosacral ligament involvement is classified as a regular or irregular hypoechogenic nodular structure, or hypoechogenic linear thickening with regular or irregular borders [27].

One sonographic indicator of rectosigmoid endometriosis is the regular or irregular hypoechogenic mass that alters and modifies the typical look of the muscle layer of the rectosigmoid wall. The rectosigmoid submucosa can be examined along the midsagittal plane as a hypoechogenic subtle difference close to a hyperechogenic stratum [32].

Applying moderate pressure on the cervical area and the lower abdomen wall to move the uterus when performing TVS helps inspect for pelvic adhesions [9]. The “sliding sign” examines how the rectum slides against the posterior uterine wall. The sign is positive when there is significant mobility between the uterus and the descendent colon [35]. A positive sign indicates a lower probability of adhesions [39]. A negative sliding sign is defined as a lack of mobility of the rectum against the uterus and the posterior vaginal fornix, indicating probable adhesion and endometriosis lesions [35,36].

Menakaya et al. discovered that the sliding sign was recognized better in the retrocervix area than in the posterior upper uterine fundus [39]. They also considered that exceeding the cut-off of 200 performed TVS provides better diagnosing for endometriosis nodules [39]. The operators with the experience of 2500 scans become proficient in performing the sliding sign technique and detecting the pouch of Douglas obliteration [39]. Except for the rectovaginal septum (RVS) DE, TVS is a precise and reliable method for non-invasive DE diagnosis [5].

In 2016, S. Guerreiro et al. formed the International Deep Endometriosis Analysis group (IDEA)-defined parameters to describe the manifestations of endometriosis and DE on TVS, fulfilling the need for established definitions in the sonographic classification and diagnosis of DE [38]. The research team formed by gynecological surgeons, robotic-assisted surgeons, and radiologists introduced a method for examining the pelvis in women suspected of having endometriosis [38]. Accordingly, TVS must be performed systematically, with endometriotic lesions measured in a standardized way, with homogeneous nomenclature to describe the DE location and specific expressions (endometriomas, adenomyosis, pelvic adhesions) [38]. Other studies have attempted to classify endometriosis lesions using ultrasonography characteristics, but none have been externally evaluated and widely adopted [35].

S. Guerreiro et al. proposes four fundamental screening ultrasound steps with suspected or confirmed endometriosis [38]. The steps can be followed in any order, as stated in the research item, with the condition that all four prove or rule out various kinds of endometriosis [38].

The four steps, as stated by S. Guerreiro, are:

1. Regular uterine and adnexal examinations (with sonographic evidence of adenomyosis or ovarian cysts).
2. Transvaginal ultrasound “soft markers” evaluation (i.e., site-specific tenderness and adnexal mobility).
3. POD status is evaluated by utilizing a real-time ultrasound “sliding sign”.
4. Examination of the anterior and posterior compartments for DE nodules [38].

The examiner’s experience impacts TVS results and reproducibility. The procedure has its own applications and limitations, but it is becoming more prevalent as a first-line diagnostic method for women suspected of endometriosis [38]. Bazot M. stated that TVS’s average accuracy in detecting DE is 85.9%, thus encouraging specialists in endometriosis to consider TVS as the first imaging method for diagnosing DE [27].

The role of ultrasound color Doppler did not prove useful in diagnosing endometriomas or DE nodules. One benefit of color Doppler is to differentiate bowel endometriosis from rectal cancer [40].

In a 2015 study, Fraser et al. compared the standard TVS with expert-guided transvaginal ultrasound (EGTV) sensitivity for endometriosis assessment [40]. They found that EGTV is more sensitive than regular pelvic ultrasound when detecting endometriosis, particularly the DE, before surgery. EGTV also contains a detailed classification of the disease’s degree and severity, which can help with surgical strategy and patient assistance [40].

#### 3.1.2. Magnetic Resonance Imaging Overview

Although MRI is a frequently used tool for diagnosing DE, there is no international agreement on the ideal imaging strategy [33]. According to local knowledge, indications and imaging techniques may differ between institutions [33].

The imaging tool with the highest overall accuracy for determining the degree of DE is magnetic resonance imaging, usually used as a second-line diagnosis method after the TVS to obtain an accurate anatomic depiction of the complete pelvic organs [33].

An MRI performed and interpreted by a specialist in endometriosis can assure the identification of DE based on the juxtaposition between normal pelvic visceral fat and endometriotic nodules or endometriomas [25]. The MRI aspect of endometriosis lesions is comparable to pelvic adhesions or fibrous tissue [41]. The radiologist’s experience in interpreting the pelvic MRI of patients suspected of endometriosis is essential in aiding the correct diagnosis of DE [42].

MRI is the imaging modality with the best average reliability for identifying the degree of DE, and has excellent sensitivity for endometriotic lesions due to its essential soft-tissue sensitivity [33]. Nonetheless, the examination and imaging interpretation should be adjusted to each patient’s concerns to attain the required accuracy [43]. Noninvasive tools demand a methodical approach to achieve consistent and comparable results [43]. When the clinical examination and TVS fail to identify lesions in symptomatic individuals, MRI gives accurate information for DE staging (particularly in parametrial lesions) [44]. When TVS is not an option, such as in cases of virgo intacta or obesity, MRI is useful. MRI can help determine the size and lateral extension of lesions before surgery, which is essential for surgical planning and approach [44].

Bruyere proposed that MRI should be conducted by radiologists who are experts in interpreting female pelvic imaging, after he studied the discrepancies in diagnostic accuracy between radiologists with different levels of experience in the MRI evaluation of DE [45]. Gynecological surgeons should recommend the patients suspected of endometriosis to imaging facilities with adequate expertise [45].

**Figure 2:**
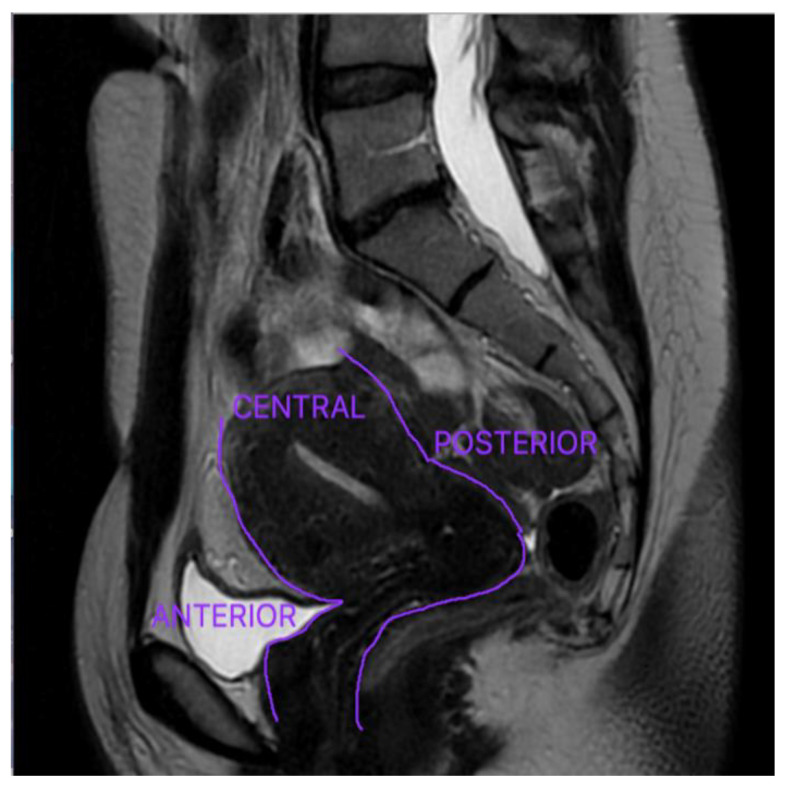
The anterior, central, and posterior pelvic compartments.

Anterior DE refers to a disease that affects the organs located in the anterior compartment: urinary bladder, the urethra, the vesicouterine pouch, and the round ligaments, and it is significantly less prevalent [20]. The uterus and ovaries are located in the central compartment, whereas the Douglas pouch, uterine torus, USL, rectum, and sigmoid colon are situated in the posterior compartment—Figure 2 [33]. DE is most typically observed in the pelvic posterior compartment, with the USL having the highest prevalence [17,46].

Lorusso et al. described a standard MRI methodology for detecting endometriosis lesions that they utilize in their center [33]. There is no agreement reached on the ideal imaging protocol worldwide. The research team illustrates the essential specifications they applied to acquire the most accurate results in detecting DE lesions in their endometriosis MRI protocol [33].

The best-quality imaging for DE nodules is acquired when using a 1.5 Tesla or 3 Tesla scanner and high-resolution phased array coils (with 8–16 channels). The TSE (turbo spin-echo)-T2w sequences must be examined in the axial, sagittal, and coronal planes with high resolution (3 mm). Regarding the endometriomas, the authors recommend using the TSE T1w (with and without fat saturation) sequences [33,44].

The detection of DE is predicated on the juxtaposition between the high signal intensity of visceral fat and the low signal level of endometriotic nodules; therefore, fat-saturated T2w images are excluded from the approach [33,47].

The protocol does not require the use of routine rectal distension [33]. However, there are benefits to rectal distention in patients with an endometriotic nodule infiltrating the rectum on standard TSE T2w imaging, which indicates the need for bowel excision [48]. The patients can undergo an MRI scan independently of the menstrual cycle phase with the condition of a full bladder [47].

Bazot et al. proposed a new series of guidelines for using MRI in diagnosing DE [48]. MRI evaluation of DE uses vaginal and rectal opacification with sonographic gel [8,48].

The instillation of intravaginal and intrarectal gel relaxes the cavities, allowing for improved visualization of the walls and potential endometriosis nodules, and determining the depth of the infiltration zone [48,49]. The gel’s contrast allows for a clearer demarcation of the peritoneal recesses (recto-vaginal and bladder-vaginal recession) [48,50]. Peristaltic artifacts are also diminished when the gel is present [48].

T2 hypointense regions attached to subsequent nodular thickening that induce anatomical distortion are often evident as adhesions [51]. Superficial endometriosis lesions are more challenging to be detected on MRI, and are best discovered intraoperatively. If the lesions are “active” with bleeding, they are seen on the peritoneal surfaces as tiny T1 hyperintensities [51].

Endometriosis ovarian cysts appear as thick-walled blood-filled tumors with uniformly high signal strength in the T1w sequence [52]. These lesions are either hyperintense or hypointense in the T2w series, or can have a typical layered look (shading sign) due to cyclic bleeding and hemosiderin deposited in time [44,52]. Endometriomas are frequently known as “chocolate cysts” [53]. Dark patches may appear within cysts in some situations in T2w sequences [52]. Atypical thickenings or vegetations should be assessed to rule out malignant progression [52].

MRI’s sensitivity and specificity for the diagnosis of endometriomas are 95% and 91%, respectively [52].

Endometrial glands and stroma are densely packed with fibro-muscular and inflammation responses in DE nodules and plaque-like lesions, which have an uneven, spiculated appearance and an MRI signal intensity comparable to that of pelvic muscles [54]. 

One of the most common locations for DE is the USL [54]. Bilateral USL lesions correlate with the existence of other posterior DE nodules, especially rectal endometriosis [55]. USL endometriosis lesions appear on the MRI sections as hypointense thickening of the ligament with regular or irregular borders [54,55]. The sensitivity and specificity of MRI in diagnosing USL endometriosis are 85% and 80%, respectively [55].

The rectum and sigmoid colon are the most common locations of intestinal DE, and the cecum and ileum are involved in approximately 5% of cases [33,50,56]. The presence of a solid or plaque-like intestinal wall thickening and disappearance of the visceral fat barrier between the rectosigmoid and the uterine wall or adnexa is used to diagnose bowel DE [33]. The supplementary signs, such as the “mushroom cap” sign, aid in the correct identification of the disease [56]. The gills of the mushroom are recreated by retractile T2 hypointense growth of the muscular stratum, whereas the mushroom cap is represented by a fine layer of T2 hyperintense submucosa and mucosa [33,56]. The sensitivity and specificity of MRI in diagnosing bowel endometriosis are 83% and 88%, respectively [57].

The” kissing ovaries” sign can be distinguished on MRI as well, in the case of ovaries collapsing in the Douglas pouch and causing pelvic adhesions. When endometriosis lesions spread from the retro-cervical space to the anterior rectum, obliteration of the Douglas pouch can be considered [57]. Small bowel movements between the uterus and the rectal wall exclude obliteration of the pouch Douglas [56,57]. The sensitivity and specificity of MRI for the assessment of endometriosis lesions of the pouch of Douglas are 89% are 94%, respectively [55].

Bladder and urinary endometriosis are uncommon and specifically affect the vesical dome, paravesical lesions, and ureteral nodules [58]. The lesions appear as single or diffuse wall thickening and signal intensity abnormalities [53]. MRI and TVS are more reliable for detecting endometriosis nodules located in the ureters as opposed to the bladder [58].

The most sensitive images for detecting ureters lesions are axial and sagittal TSE T2w images. Contrast MR urography can follow MRI to investigate the presence of ureterohydronephrosis [57]. The sensitivity of MRI for diagnosing bladder endometriosis is 88%, the specificity is 99%, and the overall diagnostic accuracy reaches 98% [55].

#### 3.1.3. Comparison between the Diagnostic Accuracy of TVS and MRI

The eight studies included in our study [8,9,10,11,12,13,14,15] compare the accuracy of the two imagistic methods in diagnosing endometriosis (Table 1).

In 2020, Zhang et al. published a meta-analysis of endometriosis diagnostic accuracy studies, revealing that TVS and MRI have high diagnostic performance in evaluating DE. TVS’ diagnostic accuracy was examined in 21 investigations, with sensitivity and specificity of 76% (95% CI (confidence interval), 67–83%) and 94%, respectively (95% CI, 88–97%). The diagnostic accuracy of MRI was examined in 13 trials, with sensitivity and specificity of 82% (95% CI, 70–90%) and 87%, respectively (95% CI, 78–92%) [59].

According to Cazalis et al., the sensitivity and specificity for ovarian endometriosis are as follows: 88.2% and 71% for TVS; 87.5% and 71% for MRI (Table 2). For uterosacral ligaments: 63% and 82.6% for TVS; 69% and 82.6% for MRI (Table 3). For rectovaginal septum: 63.2% and 100% for TVS; 47.4% and 100% for MRI (Table 4) [10].

According to Alborzi et al., the sensitivity and specificity for ovarian endometriosis are as follows: 70.86% and 92.77% for TVS; and 63.58% and 93.98% for MRI (Table 2). For uterosacral ligaments: 70.86% and 92.77% for TVS; 63.58% and 93.98% for MRI (Table 3). For bladder endometriosis: 100% and 99.6% for TVS; 100% and 99.6% for MRI (Table 5). For rectal endometriosis: 88.46% and 98.87% for TVS; and 76.92% and 96.60% for MRI (Table 6) [11].

## 4. Discussion

The objective of this review was to compile existing knowledge regarding the accuracy of endometriosis diagnostic procedures. In particular, each pelvic DE localization—ovaries, USL, rectovaginal space, bladder, and rectosigmoid—is the focus of this review, which compares the accuracy, specificity, and sensibility of TVS and MRI for each pelvic compartment.

The main strength of our study is that we focused on the most typical localization of pelvic DE to offer more support for the performance comparison of different imaging modalities. Few reviews in the specialty literature currently available assemble a larger number of DE localizations, with the majority of them concentrating on the most common ones [8,12,13,14].

A limitation was the limited number of articles included, due to our consideration of only head-to-head studies and the exclusion of some locations (vagina and anterior compartment), since there were fewer than four trials for those sites.

Regarding the diagnostic accuracy of TVS and MRI for the detection of ovarian endometriomas, the sensitivity and specificity of the two techniques were similar, with a sensitivity of 70.86–96% for TVS and 63.5–92.6% for MRI, and a specificity of 71–96% for TVS and 71–93.9%, respectively, for MRI [8,9,10,11,12,13,14,15].

Regarding the diagnostic accuracy of TVS and MRI for the detection of DE involving the rectosigmoid, the sensitivity and specificity of the two techniques were similar, with a sensitivity of 73.7–94% for TVS and 76.9–94% for MRI, and a specificity of 66.7–100% for TVS and 50–96.6%, respectively, for MRI [8,9,10,11,12,13,14,15].

Regarding the diagnostic accuracy of TVS and MRI for the detection of DE involving USL, the sensitivity of MRI was higher than TVS: 63.5–95.6% for MRI vs. 55.6–78.3% for TVS. The specificity of the two techniques was similar, 66.7–98% for TVS and 60–93.9% for MRI [8,9,10,11,12,13,14,15].

Regarding the diagnostic accuracy of TVS and MRI for the detection of DE involving the bladder, the sensitivity of MRI was higher than TVS: 33.3–100% for MRI vs. 16.7–100% for TVS. The specificity of the two techniques was similar, 98–100% for TVS and 89.5–99.6% for MRI [8,9,10,11,12,13,14,15].

Regarding the diagnostic accuracy of TVS and MRI for the detection of DE involving the rectovaginal space, the sensitivity of MRI was higher than TVS: 47.4–95.2% for MRI vs. 9–86.3% for TVS. The specificity of the two techniques was similar: 88.9–100% for TVS and 71.1–100% for MRI [8,9,10,11,12,13,14,15].

Pelvic deep endometriosis can be harder to identify preoperatively because of its significant clinical variability. According to existing data, the condition should be evaluated non-invasively by integrating knowledge from the patient’s medical history, clinical exam, imaging techniques, and therapy outcome. Based on all information acquired through the studies cited above, expert-guided TVS is a more accurate investigation than standard pelvic ultrasound in the assessment of DE [40]. TVS should continue to be the primary line of diagnosis for patients suspected of having deep pelvic endometriosis [11,18]. Moreover, it contains a thoroughly segmented depiction of the condition’s invasion and severity, which can help in surgical planning and patient education [32]. TVS is highly operator-dependent, and effective diagnostic outcomes can only be attained by a well-trained, skilled medical team [32,40].

TVS is superior for diagnosing endometriosis cysts, and MRI is better used for diagnosing torus, uterosacral ligaments, and intestinal and bladder DE lesions [10,60,61]. MRI should be the preferred imaging method for finding intestinal or rectal DE and rectovaginal septum DE, following various research [9,34].

MRI has established itself as a helpful diagnostic tool, and is the ideal preoperatively imaging technique for detecting DE lesions [44,62]. As a physician and endometriosis specialist, it is important to assist radiologists to acquire relevant images using a personalized MRI acquisition technique, and to detect a large spectrum of pelvic alterations that may develop from endometriosis, since the detection rate of MRI varies based on radiologist experience [33,44].

MRI should be reserved for equivocal TVS findings in rectovaginal or vesical endometriosis [11,31]. The first imaging test for patients suspected of endometriosis should be TVS. MRI should be combined with TVS for all the patients suffering from unidentified pelvic discomfort [10,13]. However, MRI is not a routine investigation in all patients suspected of endometriosis. The gynecologist must request an MRI investigation when the TVS is insufficient or cannot be performed. The methods depend on the experience of the gynecologist and radiologist in making a preoperative assessment of the lesions, so the surgical treatment is performed in the most complete way possible.

DE lesions are more frequently identified preoperatively by endometriosis specialists with a substantial background in the management and therapy of the condition [63,64]. A gynecologist who has been taught and has expertise with TVS can identify endometriosis lesions more accurately and effectively than a gynecologist who does not generally interpret endometriosis lesions and cannot integrate the imaging interpretations for the correct diagnosis [41]. When diagnosing DE, the examiner’s experience is the most crucial factor to consider.

Early identification of DE should be accompanied by a precise assessment of the disease’s severity, which can aid the planning of the surgical intervention [64,65]. Accurate preoperative examinations can help decrease the number of surgeries that are incomplete due to a lack of knowledge of the disease’s width and complexity, as well as direct recommendations to expert centers. The adequate surgical removal of endometriosis lesions (parametrial, uterosacral ligaments, intestinal nodules) has been linked to increased quality of life [66,67,68]. There have been documented benefits in the overall health, quality of life, and psychological state of patients with endometriosis after surgical laparoscopic treatment [67,68]. When compared to hormonal treatments, laparoscopic excision of endometriosis lesions produces better results and fewer adverse effects [67].

## 5. Conclusions

The two imaging exams are best used together for evaluating DE lesions prior to surgery.

Despite the limited accuracy and precision, bimanual examination and analysis of clinical manifestations should not be discounted as an important diagnostic means in detecting DE and establishing subsequent treatment procedures. In combination with TVS, the bimanual vaginal examination may aid in determining the extent and depth of the DE lesions.

By comparing the two main imaging techniques, we emphasized the necessity for a harmonized, preoperatively noninvasive diagnosis protocol. To appropriately diagnose DE, the protocol should be parted by the three pelvic compartments (anterior, middle, and posterior). The physician should decide between the two imaging methods or utilize both based on the patient’s clinical examination and symptoms.

We suggest using a uniform methodology in future investigations, as proposed by the IDEA consensus [54] or S. Guerreiro [38], regarding the TVS methodology for detecting endometriosis lesions, and Lorusso [33] and Bazot [48], regarding MRI methodology, in order to reduce variation.

An accurate preoperative DE assessment is mandatory for proper surgical planning. Direct referrals to specialized centers or experts are required, especially for deep endometriosis that necessitates a multidisciplinary approach.

## Figures and Tables

**Table 1:** Comparison between the diagnostic accuracy of transvaginal sonography and magnetic resonance imaging in DE as reported in different studies.

Reference	Number of Patients	Locations of DE	Imaging Techniques	Mean Age	Year of Publication
Hudelist et al. [9]	126	Ovaries, uterosacral ligaments, rectovaginal space, bladder, rectosigmoid	TVS	32.2	2011
Cazalis et al. [10]	25	Ovaries, uterosacral ligaments, rectovaginal space, bladder, rectosigmoid	TVS + MRI	35.4	2012
Alborzi et al. [11]	317	Ovaries, uterosacral ligaments, rectovaginal space, bladder, rectosigmoid	TVS + MRI	31	2018
Bazot et al. [12]	92	Uterosacral ligaments, rectovaginal space, rectosigmoid	TVS + MRI	31.8	2009
Idrielle et al. [15]	49	Uterosacral ligaments, rectovaginal space, bladder, rectosigmoid	TVS + MRI	-	2019
Kruger et al. [14]	152	Uterosacral ligaments, ovaries, bladder, rectosigmoid	MRI	33.5	2013
Saccardi et al. [8]	102	Uterosacral ligaments, rectovaginal space	TVS + MRI	32.3	2012
Saba et al. [13]	30	Ovaries, uterosacral ligaments, rectosigmoid	MRI	34	2011

**Table 2:** The diagnostic accuracy of TVS and MRI in ovarian endometriosis (as reported in different studies).

Ovaries	TVS		MRI	
	Sensitivity	Specificity	Sensitivity	Specificity
Hudelist et al. [9]	96%	96%	-	-
Cazalis et al. [10]	88.2%	71%	87.5%	71%
Alborzi et al. [11]	70.86%	92.7%	63.5%	93.9%
Saba et al. [13]	-	-	92.6%	91.3%
Kruger et al. [14]	-	-	86.3%	73.6%

**Table 3:** The diagnostic accuracy of TVS and MRI in uterosacral ligaments DE (as reported in different studies).

USL	TVS		MRI	
	Sensitivity	Specificity	Sensitivity	Specificity
Hudelist et al. [9]	63%	98%	-	-
Cazalis et al. [10]	63%	82.6%	69%	82.6%
Alborzi et al. [11]	70.8%	92.7%	63.5%	93.9%
Bazot et al. [12]	78.3%	66.7%	84.4%	88.9%
Idrielle et al. [15]	74%	67%	94%	60%
Kruger et al. [14]	-	-	77%	68%
Saccardi et al. [8]	55.6%	95.6%	95.6%	75%
Saba et al. [13]	-	-	80%	84.6%

**Table 4:** The diagnostic accuracy of TVS and MRI in rectovaginal DE (as reported in different studies).

Rectovaginal Space	TVS		MRI	
	Sensitivity	Specificity	Sensitivity	Specificity
Hudelist et al. [9]	64%	99%	-	-
Cazalis et al. [10]	63.2%	100%	47.4%	100%
Alborzi et al. [11]	86.3%	94.8%	95.2%	71.1%
Bazot et al. [12]	9%	98.7%	54.5%	98.7%
Idrielle et al. [15]	67%	100%	83%	93%
Saccardi et al. [8]	63.9%	88.9%	83.3%	77.8%

**Table 5:** The diagnostic accuracy of TVS and MRI in urinary bladder DE (as reported in different studies).

Bladder	TVS		MRI	
	Sensitivity	Specificity	Sensitivity	Specificity
Hudelist et al. [9]	50%	98%	-	-
Cazalis et al. [10]	16.7%	100%	33.3%	89.5%
Alborzi et al. [11]	100%	99.6%	100%	99.6%
Idrielle et al. [15]	89%	100%	100%	95%
Kruger et al. [14]	-	-	81%	94%

**Table 6:** The diagnostic accuracy of TVS and MRI in rectosigmoid DE (as reported in different studies).

Rectosigmoid	TVS		MRI	
	Sensitivity	Specificity	Sensitivity	Specificity
Hudelist et al. [9]	90%	99%	-	-
Cazalis et al. [10]	73.7%	66.7%	89.5%	50%
Alborzi et al. [11]	88.4%	98.8%	76.9%	96.6%
Bazot et al. [12]	93.6%	100%	87.3%	93.1%
Idrielle et al. [15]	94%	84%	94%	84%
Kruger et al. [14]	-	-	80%	77%
Saba et al. [13]	-	-	73.9%	83.9%

## Data Availability

Not applicable.
